# Supplementary material for: LISA Pathfinder Performance Confirmed in an Open-Loop Configuration: Results from the Free-Fall Actuation Mode
Source: Phys Rev Lett. Author manuscript; Available in PMC 2021 Jan 15. (PMC7810161; doi:10.1103/PhysRevLett.123.111101)
Supplement: Supplemental File [file NIHMS1542524-supplement-Supplemental_File.pdf]

Additional Material to "LISA Pathfinder Performance Confirmed in an Open-Loop Configuration: Results from the Free-Fall Actuation Mode"

LISA Pathfinder Collaboration

BHGZ METHOD IMPLEMENTATION

The decimation of free-fall data considers an integer factor of the number of samples per experimental time, N_{exp}, such that each experimental segment still contains, after decimation, a fixed number of data points. In our case, with data originally sampled at 10 Hz and $T_{\mathrm{exp}} = 350.2\,\mathrm{s}$, $N_{\mathrm{exp}} = 3502$. The decimation factor applied is equal to 103 which gives 34 samples per experimental time, n_{tot}, with sampling time $T_{\mathrm{samp}} \sim 10.3\,\mathrm{s}$.

For the analysis, we remove $T_{\mathrm{cut}} = 2\,\mathrm{s}$ of data at the beginning and at the end of each flight in order to avoid transients which may be close to the kicks. The low-pass filter length, T_{win}, is set up in such a way as to have an integer number of finite windows per flight time, T_{flight}:

$$T_{\mathrm{win}} = T_{\mathrm{flight}} - 2T_{\mathrm{cut}} - (n_{\mathrm{keep}} - 1)T_{\mathrm{samp}} \tag{1}$$

where n_{keep} is the number of samples maintained per flight time. With 34 samples per experimental time, divided into 25 samples in the flight time and 9 overlapping with the impulse which are set to zero, the filter length is equal to 98 s when $T_{\mathrm{imp}} = 1\,\mathrm{s}$ and 94 s when $T_{\mathrm{imp}} = 5\,\mathrm{s}$.

REMARKS ON SPECTRAL ESTIMATION

According to the modified Welch periodogram method [1], the mean value of the power spectral density (PSD), at each discrete time frequency $\phi_k \equiv k\,2\pi/N$, of a discrete time, zero-mean stochastic process, $x[\mathrm{n}]$, is:

$$\begin{aligned}\langle S_k \rangle &= \frac{1}{N}\sum_{\mathrm{m,n}=0}^{N-1} \langle x[\mathrm{n}]\,x[\mathrm{m}]\rangle\, w[\mathrm{n}]\,w[\mathrm{m}] e^{-ik\frac{2\pi}{N}(\mathrm{n-m})} \\ &= \frac{1}{N}\sum_{\mathrm{m,n}=0}^{N-1} R_x[\mathrm{n-m}] w[\mathrm{n}]\,w[\mathrm{m}] e^{-ik\frac{2\pi}{N}(\mathrm{n-m})},\end{aligned} \tag{2}$$

where N is the number of samples per data stretch (or periodogram), $w[n]$ is the normalized spectral window applied to the periodogram to ensure that it smoothly approaches zero at its ends [1]; within the LPF collaboration, the minimum "4-term Blackman-Harris window" (BH92) is used. $R_x[\mathrm{n-m}] \equiv \langle x[\mathrm{n}]\,x[\mathrm{m}]\rangle$, is the autocorrelation of $x[\mathrm{n}]$, which is defined, according to the Wiener-Khinchin theorem, as the inverse Fourier transform of the PSD of the *infinite length* $x[\mathrm{n}]$ series, $S_x(\phi)$:

$$R_x[\mathrm{n-m}] \equiv R_{\mathrm{n,m}} = \frac{1}{2\pi}\int_{-\pi}^{\pi} S_x(\phi) e^{i\phi(\mathrm{n-m})} d\phi. \tag{3}$$

Defining the matrix:

$$\gamma_{k,\mathrm{m}} = \frac{1}{\sqrt{N}} w[\mathrm{m}] e^{-ik\frac{2\pi}{N}\mathrm{m}}, \tag{4}$$

we can express the PSD estimate in the following matrix form:

$$\langle S_k \rangle = \sum_{\mathrm{n,m}=0}^{N-1} \gamma_{k,\mathrm{n}} R_{\mathrm{n,m}} \gamma^{\dagger}_{m,\mathrm{k}} = [\mathrm{diag}(\gamma \cdot R \cdot \gamma^{\dagger})]_k. \tag{5}$$

The k-mean value of the spectrum is thus given by the triple matrix product of Eq. 5.

BIAS REMOVAL ALGORITHM

The gaps in the free-fall measurement data can bias the spectral estimate, especially at low frequencies ($\leq$ 1 mHz). This effect can be calculated and removed by means of an *a-posteriori* approach. It is possible to rewrite Eq. 2 using Eq. 3 to obtain:

$$\langle S_k \rangle = \frac{1}{2\pi}\int_{-\pi}^{\pi} S_x(\phi) \left| h\left(\phi - k\frac{2\pi}{N}\right)\right|^2 d\phi, \tag{6}$$

where the window $h(\phi)$ is defined as:

$$h(\phi) = \frac{1}{\sqrt{N}} \sum_{\mathrm{n}=0}^{N-1} w[\mathrm{n}] \mathrm{e}^{-\mathrm{i}\phi\mathrm{n}}, \tag{7}$$

in other words, $h(\phi)$ is the Discrete Time Fourier Transform of the window: $(1/\sqrt{N})\,\Theta[\mathrm{n}]\,\Theta[\mathrm{N\text{-}1\text{-}n}] w[\mathrm{n}]$, where $\Theta[\mathrm{n}]$ is the Heaviside theta function. Thus, Eq. 6 shows that the PSD estimate is the result of the action of $h(\phi)$ on the "true" PSD, $S_x(\phi)$. In the case of the "Blackman-Harris Gap Zero" (BHGZ) method, explained in the paper to which this supplemental material refers to, $w[\mathrm{n}]$ is the result of the multiplication of the standard BH92 spectral window with a rectangular-wave with period T_{exp} and duty cycle $n_{\mathrm{keep}}/n_{\mathrm{tot}}$, used to set the gaps to zero. In other words, $w[\mathrm{n}]$ is a BH92 window containing zeros at the positions of the kicks.

As shown in [2], the multiplication of data for a rectangular-wave essentially down-converts noise at harmonic multiples of T_{exp}, producing an aliasing effect. For the specific case of the white noise spectral component, an analytic calculation reported in [2] demonstrates that, to get rid of the lack of points set to zero, the spectrum must be multiplied by a normalization factor equal

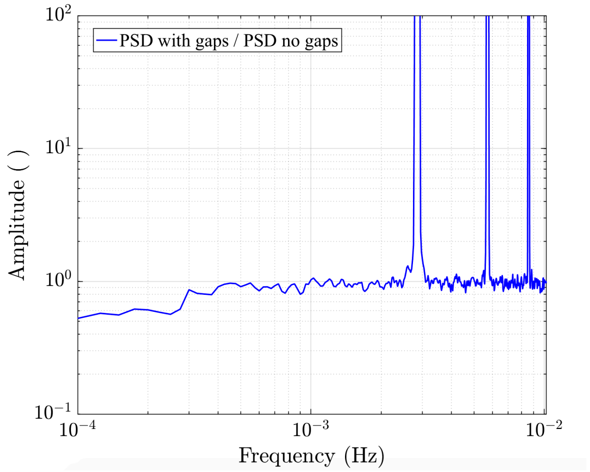

Figure 1. Ratio between the PSD of noise data measured in continuous control in December 2016, which has been analyzed with the BHGZ technique, gapped and multiplied by $n_{\rm tot}/n_{\rm keep}$, and the PSD of the same data, filtered and decimated only.

to the inverse of the duty cycle of the experiment (i.e. $n_{\rm tot}/n_{\rm keep}$).

To have an idea of the effect of gaps on the other spectral noise contributions, let us consider an estimate of Δg measured with continuous control along x, analyze it with the BHGZ technique and insert artificial gaps of the same duration and repetition rate as those in the free-fall mode experiment. If we divide the resulting spectrum, corrected with the normalization factor defined above, by the spectrum of the same data, decimated and filtered only, we obtain the result depicted in Fig. 1. As shown, gaps cause an underestimation of the spectrum at frequencies below 1 mHz, while spikes are visible at multiple frequencies of the experimental one (~ 2.8 mHz).

Because of the non-invertibility of Eq. 6, the estimation of the spectral bias due to gaps, beyond the white noise contribution, is based on a "pseudo-inverse" approach: we look for the optimal shape of $S_x(\phi)$ that, through the action of the overall window, reproduces the estimated PSD, $\langle S_k \rangle$. In practice, we assume that the PSD of Δg is composed of various contributions, the combination of which gives a continuous spectrum that, when passed through our analysis process, is expected to match the calculated gapped-data spectrum. In the following we will go through the steps of this procedure.

First, we define a continuous model for the spectrum. Since we want to correct effectively the PSD at low frequency, the model is a linear combination of the two noise contributions arising at frequency below ~ 30 mHz [3]:

$$S_{mod} \simeq \alpha_w S_w + \alpha_{1/f^2} S_{1/f^2}, \tag{8}$$

where:

- S_w refers to a frequency-independent component (white noise) of the spectrum, which dominates in the [1, 30] mHz frequency range.
- S_{1/f^2} is defined as:

$$S_{1/f^2}(f) = \frac{1}{2}\frac{1}{1+\frac{f^2}{f_0^2}},$$

with a roll-off frequency, f_0, of 1 μHz after which it decays as $1/f^2$.

and α_w, α_{1/f^2} are the free parameters in the fit. It is worth noting that the result is independent on the choice of the roll-off frequency of the S_{1/f^2} term.

Then, the model is transformed according to what is performed on free-fall mode data. In practice, we compute, for both spectral terms, the corresponding autocorrelation function and evaluate the matrix product as in Eq. 5. Indeed, it is convenient, for numerical reasons, to look for the best shape of R, instead of $\langle S \rangle$, that better fits the data (see Eq. 5). In reality, since data are decimated and filtered, the autocorrelations must be first convolved with the impulse response of the BH low-pass filter, h_{filt}, as follows:

$$R_{filt}[\mathrm{m}] = (h_{filt} * R * h_{filt})_{\mathrm{m}\times N_d}, \tag{9}$$

where $*$ indicate discrete convolution and N_d is the decimation factor we apply to analyze the free-fall mode data. The model to which we fit data is thus the following:

$$\begin{aligned} S^{gap}_{mod} &= \alpha_w[\mathrm{diag}(\gamma \cdot R_{filt,w} \cdot \gamma^\dagger)] \\ &+ \alpha_{1/f^2}[\mathrm{diag}(\gamma \cdot R_{filt,1/f^2} \cdot \gamma^\dagger)], \end{aligned} \tag{10}$$

where the γ matrices, defined in Eq. 4, contain the "gapped" spectral window, w[n], applied on data (we omit, for simplicity, the matrix indices).

The linear least-squares fit is performed in frequency domain iteratively, each time assuming a theoretical uncertainty based on the PSD estimate and using the fit coefficients obtained at the preceding iteration. The frequency range considered is $[0.1, 10]$ mHz, where one bin every four is used to avoid correlated data [4], while the peaks are discarded from the fit. The resulting number of degrees of freedom is 63, with 10 iterations for the fit.

BIAS REMOVAL ON FREE-FALL MODE DATA MEASURED IN DECEMBER

The results of the bias removal procedure on the free-fall mode data measured in December 2016, are shown in Fig. 2. The red dashed line in Fig. 2a is obtained from the best fit to the experimental gapped ASD of Δg, which is marked in blue. In practice, it is the result of Eq. 10 when the best fit values are used for the α_i coefficients. The values of the fit coefficients are reported in Table I.

It must be stressed that the values of the fit coefficients depend on the numerical method and thus they do not have any physical significance, instead, the physical result is independent on the method used to fit data.

Parameter	value $\mathrm{fm/s^2/\sqrt{Hz}}$	error	χ^2
$\sqrt{\alpha}_w$	2.97	0.04	1.4
$\sqrt{\alpha}_{1/f^2}$	1.09	0.09	

Table I. Values of the fit coefficients and reduced χ^2 obtained by fitting the spectrum of the free-fall mode experiment, carried out in December 2016, to the model of Eq. 10.

Using the fit coefficients, we can extract the Δg ASD without gaps using Eq. 10 again, but with $w[\mathrm{n}]$ *continuous* instead of gapped. The result is marked in dash-dotted line in Fig. 2(a). In other words, the dash-dotted line is an estimate of the "native" ASD of Δg measured in the free-fall mode experiment, which converts into the dashed line when gaps are inserted. The ratio between the two spectra, which assumes the functional form of the model in Eq. 8 but is independent on the amplitudes of the various terms, corresponds to the bias introduced by gaps. We can thus remove it from the Δg ASD of the free-fall mode data, as explained in the paper to which this supplemental material refers. Fig. 2(c) depicts the ASD of free-fall data before (thin line) and after the bias removal (thick line), where the latter has been normalized for the transfer function of the BH filter used. Note that, since the peaks are not fitted, they remain in the final spectrum, as visible in the figure. Finally, the line in Fig. 2(b) results from the subtraction of the ratio between the solid and the dashed spectra of Fig. 2(a), by 1. The difference between the distributions of the two above mentioned spectra, is significant at the 5% level, according to the Kolmogorov-Smirnov test [5].

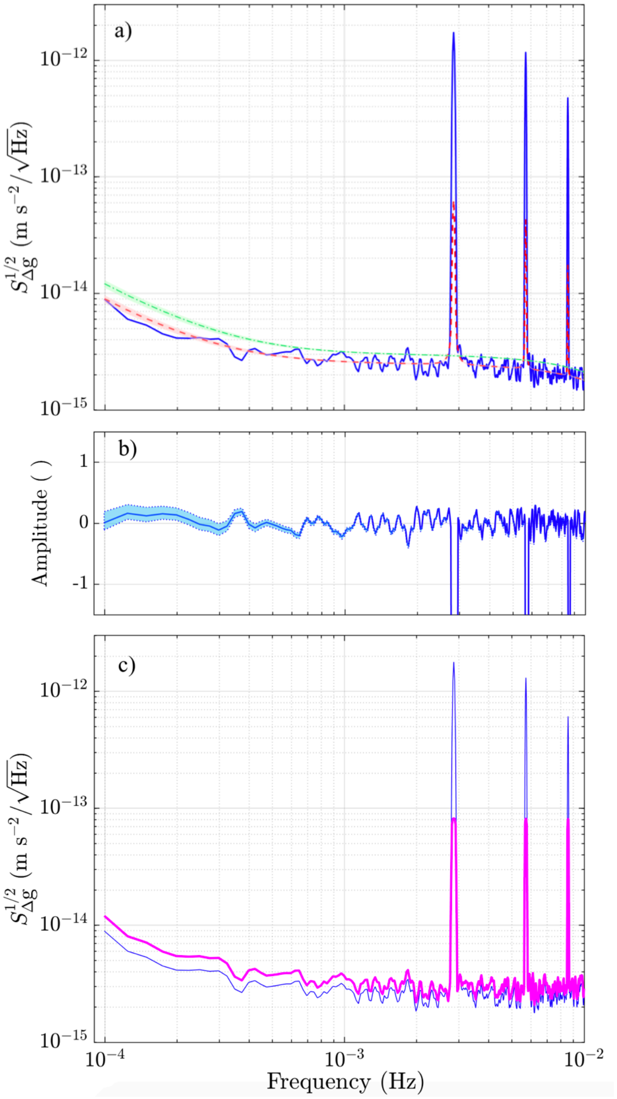

Figure 2. Bias removal procedure applied on data of free-fall mode measured in December 2016 (see the text for details).

CALIBRATION OF THE METHOD WITH CONTINUOUS NOISE DATA

In order to test the accuracy of the bias removal algorithm, we have applied it on noise data measured in continuous actuation mode with gaps inserted artificially, as described in the first section. The final result, corrected for the bias, is compared with the original ASD of Δg sampled at 10 Hz in Fig. 3. The figure includes also a similar test where an additional window, namely an Hann window, is applied to data between the gaps such that they smoothly approach zero at their ends. The resulting fit coefficients are collected in Table II.

As visible in Fig. 3, in both cases we achieve 1σ agreement between the ASDs of the unbiased gapped data and that of the original-continuous data at frequencies below 1 mHz and this result is confirmed by the agreement in the estimate of the α_{1/f^2} coefficient.

FINAL CONSIDERATIONS

We noted that the spectral leakage due to gaps, arising from high towards low frequencies, is limited in free-fall data thanks to the low noise level of the interferometer measured on board LPF, which is well below the requirements [7]. Indeed, gaps do not cause a severe aliasing of the spectrum in the LPF sensitivity band but rather they induce an underestimation of the noise power which is related to the duty cycle of the rectangular-wave used to gap the data. Nevertheless, a sanity check of the data analysis method has been performed using LPF data with interferometer readout noise increased artificially by a factor ~ 100. Also in this case, the method has allowed to remove the spectral bias effectively at low frequencies.

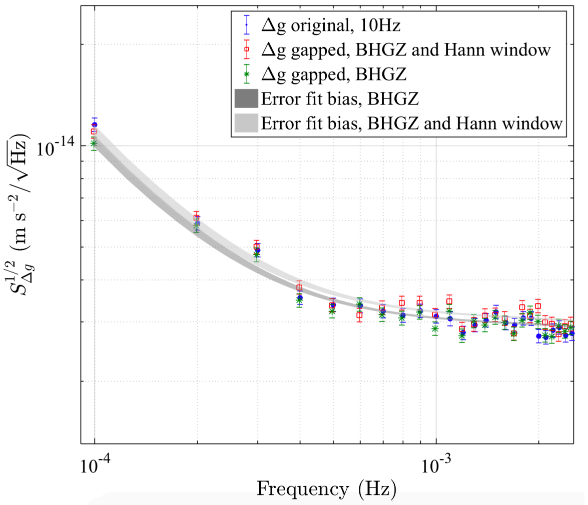

Figure 3. Calibration tests using continuous noise data. The dots depict the original Δg ASD measured in December 2016 with continuous actuation mode (78 periodograms). The asterisks indicate the result of applying the BHGZ technique to the previous data with synthetic gaps set to zero and with bias removed. Finally, the squares show the same analysis when an Hann window is applied on data between gaps only. The errors are calculated as described in [6].

Test	Parameter	value	error	χ^2
		fm/s^2/$\sqrt{\mathrm{Hz}}$		
BHGZ	$\sqrt{\alpha_w}$	2.95	0.02	1.06
	$\sqrt{\alpha_{1/f^2}}$	0.92	0.04	
BHGZ and Hann	$\sqrt{\alpha_w}$	3.07	0.02	1.05
	$\sqrt{\alpha_{1/f^2}}$	0.99	0.05	

Table II. Fit results of the two calibration tests using the continuous control Δg estimate measured in December 2016.

[1] P. Welch, IEEE Transactions on Audio and Electroacoustics **15**, 70 (1967).

[2] G. Russano, A. Cavalleri, A. Cesarini, R. Dolesi, V. Ferroni, F. Gibert, R. Giusteri, M. Hueller, L. Liu, P. Pivato, H. B. Tu, D. Vetrugno, S. Vitale, and W. J. Weber, Classical and Quantum Gravity **35**, 035017 (2018).

[3] M. Armano *et al.*, Phys. Rev. Lett. **120**, 061101 (2018).

[4] S. Vitale, G. Congedo, R. Dolesi, V. Ferroni, M. Hueller, D. Vetrugno, W. J. Weber, H. Audley, K. Danzmann, I. Diepholz, M. Hewitson, N. Korsakova, L. Ferraioli, F. Gibert, N. Karnesis, M. Nofrarias, H. Inchauspe, E. Plagnol, O. Jennrich, P. W. McNamara, M. Armano, J. I. Thorpe, and P. Wass, Phys. Rev. D **90**, 042003 (2014).

[5] L. Ferraioli, G. Congedo, M. Hueller, S. Vitale, M. Hewitson, M. Nofrarias, and M. Armano, Phys. Rev. D **84**, 122003 (2011).

[6] M. Armano *et al.*, Supplemental Material, Phys. Rev. Lett. **120**, 061101 (2018), section on Bayesian ASD estimate.

[7] M. Armano *et al.*, Phys. Rev. Lett. **116**, 231101 (2016).
